# Improved Detection of Small (<2 cm) Hepatocellular Carcinoma via Deep Learning-Based Synthetic CT Hepatic Arteriography: A Multi-Center External Validation Study

**DOI:** 10.3390/diagnostics16020343

**Published:** 2026-01-21

**Authors:** Jung Won Kwak, Sung Bum Cho, Ki Choon Sim, Jeong Woo Kim, In Young Choi, Yongwon Cho

**Affiliations:** 1 Department of Radiology, Anam Hospital, College of Medicine, Korea University, 73 Goryeodae-ro, Seongbuk-gu, Seoul 02841, Republic of Korea; jwkwak@korea.ac.kr (J.W.K.); kspringsim@korea.ac.kr (K.C.S.); 2 Department of Radiology, Guro Hospital, College of Medicine, Korea University, 148 Gurodong-ro, Guro-gu, Seoul 08308, Republic of Korea; pridebio@naver.com; 3 Department of Radiology, Ansan Hospital, College of Medicine, Korea University, 123 Jeokgeum-ro, Danwon-gu, Ansan-si 15355, Republic of Korea; ciy150244@gmail.com; 4 Department of Computer Science and Engineering, Soonchunhyang University, Asan 31538, Republic of Korea

**Keywords:** hepatocellular carcinoma, deep learning, generative adversarial networks, CT hepatic arteriography, detection

## Abstract

**Background/Objectives:** Early detection of hepatocellular carcinoma (HCC), particularly small lesions (<2 cm), which is crucial for curative treatment, remains challenging with conventional liver dynamic computed tomography (LDCT). We aimed to develop a deep learning algorithm to generate synthetic CT during hepatic arteriography (CTHA) from non-invasive LDCT and evaluate its lesion detection performance. **Methods:** A cycle-consistent generative adversarial network with an attention module [Unsupervised Generative Attentional Networks with Adaptive Layer-Instance Normalization (U-GAT-IT)] was trained using paired LDCT and CTHA images from 277 patients. The model was validated using internal (68 patients, 139 lesions) and external sets from two independent centers (87 patients, 117 lesions). Two radiologists assessed detection performance using a 5-point scale and the detection rate. **Results:** Synthetic CTHA significantly improved the detection of sub-centimeter (<1 cm) HCCs compared with LDCT in the internal set (69.6% vs. 47.8%, *p* < 0.05). This improvement was robust in the external set; synthetic CTHA detected a greater number of small lesions than LDCT. Quantitative metrics (structural similarity index measure and peak signal-to-noise ratio) indicated high structural fidelity. **Conclusions:** Deep-learning–based synthetic CTHA significantly enhanced the detection of small HCCs compared with standard LDCT, offering a non-invasive alternative with high detection sensitivity, which was validated across multicentric data.

## 1. Introduction

Hepatocellular carcinoma (HCC) is a leading cause of cancer-related mortality worldwide, with patient prognosis relying heavily on the stage at diagnosis. Although curative modalities such as surgical resection, liver transplantation, and thermal ablation are highly effective, their success is strictly confined to patients with single, small nodules (<2 cm) and preserved liver function [1,2,3,4,5,6,7]. Consequently, precise and early detection of sub-centimeter lesions constitutes the cornerstone of improving long-term survival.

The current international guidelines, including those formulated by the American Association for the Study of Liver Diseases and European Association for the Study of the Liver, advocate for surveillance using liver dynamic computed tomography (LDCT) or magnetic resonance imaging (MRI) in high-risk cohorts [8,9]. The definitive diagnosis typically hinges on identifying the hallmark hemodynamic pattern: arterial phase hyperenhancement, followed by “washout” in the portal venous or delayed phases. However, the sensitivity of standard LDCT is frequently suboptimal for observing minute lesions [10]. Previous studies have indicated that diagnostic sensitivity drops precipitously for sub-centimeter HCCs compared with larger tumors, as these minute lesions often fail to exhibit distinct arterial hypervascularity due to rapid blood flow, partial volume effects, or temporal mismatches in contrast bolus timing [11,12]. Despite the formidable technical challenges associated with identifying sub-centimeter nodules, early detection is paramount because it facilitates timely curative intervention, ultimately yielding superior overall survival outcomes [13].

CT during hepatic arteriography (CTHA) has traditionally served as the reference standard for detecting such elusive hypervascular HCCs [14,15,16]. By delivering contrast media directly into the hepatic artery, CTHA achieves a significantly higher iodine concentration within the tumor relative to the parenchyma, thereby maximizing tumor-to-liver contrast. While studies have consistently demonstrated the superior yield of CTHA over that of intravenous LDCT or MRI [15,16], its invasive nature limits clinical application. Consequently, very few institutions worldwide continue to perform CTHA, which explains the scarcity of recent large-scale studies utilizing actual CTHA datasets. Even at our institution, CTHA is selectively performed in complex cases where lesion characterization remains inconclusive via cone-beam CT or conventional angiography during transarterial chemoembolization (TACE). This clinical rarity underscores the significance of our study: by developing a deep-learning–based synthetic CTHA model, we aim to provide the superior detection benefits of CTHA through a non-invasive, routine CT protocol, thereby overcoming the practical barriers posed by invasive procedural requirements.

To bridge the gap between the detection superiority of CTHA and the non-invasive accessibility of LDCT, we adopted a deep-learning–based domain adaptation approach. Although recent advances in generative models, including diffusion probabilistic models, have garnered attention, generative adversarial networks (GANs) remain a robust framework for medical image translation because of their computational efficiency and ability to preserve high-frequency structural details [17,18,19,20]. Specifically, the CycleGAN architecture facilitates unsupervised mapping between two domains without the requirement for perfectly paired data. However, standard CycleGANs are prone to “mode collapse” or geometric distortions, which are unacceptable in clinical diagnostics where anatomical fidelity is paramount [21]. To mitigate these limitations, we employed Unsupervised Generative Attentional Networks with Adaptive Layer-Instance Normalization (U-GAT-IT). This architecture integrates an attention module that explicitly guides the model to focus on discriminative regions, specifically hypervascular foci, whereas the adaptive normalization function preserves the underlying morphological integrity of the liver parenchyma.

We postulated that a U-GAT-IT model, trained on unpaired LDCT and CTHA datasets, can learn the complex non-linear mapping of contrast enhancement, thereby generating “synthetic CTHA” images directly from non-invasive LDCT scans. Unlike previous studies that focused primarily on technical image quality metrics or single-center feasibility, this study aimed to (1) establish a robust algorithm for synthetic CTHA generation that maintains anatomical precision, (2) evaluate its clinical utility in improving the detection of small HCCs (<2 cm), and (3) validate its generalizability and robustness using a multicenter external validation cohort.

## 2. Materials and Methods

### 2.1. Study Population and Patient Selection

#### 2.1.1. Confirmation of HCC Diagnosis

The diagnosis of all included lesions was established based on rigorous clinicoradiological criteria. For lesions with typical imaging features, HCC was diagnosed according to the current international practice guidelines. For sub-centimeter (<1 cm) lesions with atypical radiological features, retrospective diagnostic confirmation was performed by evaluating the lipiodol tagging status on postprocedural CT scans and/or by documenting significant interval size growth and hemodynamic changes during a minimum 12-month longitudinal follow-up with serial CT or MRI.

We reviewed the medical records of patients diagnosed with HCC who underwent TACE as an initial treatment between January 2010 and January 2022. A total of 493 potentially eligible patients were identified for training and internal validation and 94 potentially eligible patients were identified for external validation.

#### 2.1.2. Study Design and Ethical Consideration

This multicenter retrospective study was conducted in accordance with the Declaration of Helsinki and approved by the Institutional Review Boards (IRB) of Korea University Anam (2025AN0155), Guro (2023GR0131), and Ansan (2023AS0116) Hospitals. The requirement for informed consent was waived due to the retrospective nature of the analysis.

#### 2.1.3. Training and Internal Validation Cohorts

**Figure 1:**
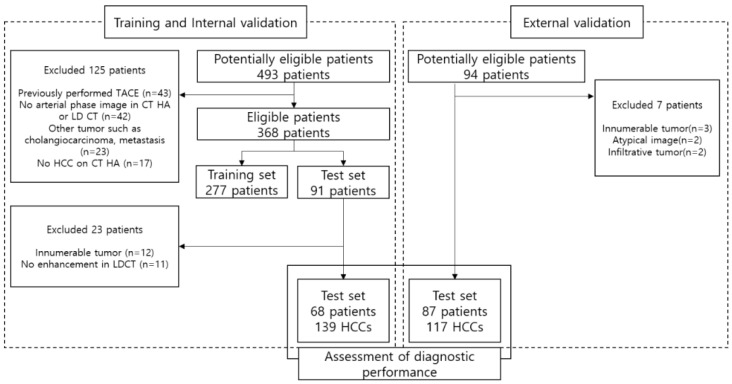
Flow diagram of patient selection. A total of 493 potentially eligible patients were initially screened. After applying inclusion and exclusion criteria, 277 patients were selected for the training set. The model was validated using two independent datasets: an internal validation set of 68 patients (139 HCCs) and an external validation set of 87 patients (117 HCCs) from two separate institutions.

We searched the medical records of patients diagnosed with HCC who underwent TACE as initial treatment at Korea University Anam Hospital between January 2010 and January 2022. Initially, a pool of 493 eligible patients was identified. Patient eligibility was determined based on stringent inclusion and exclusion criteria (Figure 1).

The inclusion criteria mandated that patients must have: (1) undergone both CTHA and LDCT examinations within a 3-month interval prior to TACE to ensure temporal consistency; (2) had a confirmed diagnosis of HCC based on typical radiological features, specifically, arterial enhancement followed by washout, in accordance with established clinical guidelines [8,9]; (3) for small (<1 cm) hypervascular lesions with equivocal radiologic features, inclusion was predicated on the presence of definitive lipiodol uptake on postprocedural lipiodol CT scans. In cases where lipiodol tagging was indistinct, lesions were confirmed to be HCC only after demonstrating significant interval growth over a longitudinal follow-up period of at least 12 months via serial CT or MRI surveillance.

Conversely, patients were excluded if they exhibited: (1) a history of prior TACE or dense lipiodol retention, which could confound image analysis due to beam-hardening artifacts; (2) concurrent hepatic malignancies such as cholangiocarcinoma, combined HCC-cholangiocarcinoma, or metastasis; (3) an absence of identifiable enhancing tumors on the reference CTHA images (ground truth); (4) diffuse infiltrative patterns or extensive multifocal disease (>10 nodules) that rendered individual lesion quantification unreliable; or (5) suboptimal image quality resulting from severe motion artifacts.

Following the application of these eligibility criteria, 277 patients were allocated to the training set. Subsequently, an independent internal validation set was constructed, comprising 68 patients (with 139 HCC lesions) who were distinct from the training cohort (Table 1).

#### 2.1.4. External Validation Cohort

To evaluate the generalizability of the proposed model rigorously and ensure its robustness against domain shifts caused by different scanner protocols, we curated an independent external validation set. The cohort comprised 87 patients (117 HCC lesions) recruited from two separate institutions (Korea University Guro Hospital and Ansan Hospital) between January 2019 and December 2021. This multi-institutional design was specifically intended to verify the clinical applicability of the synthetic CTHA generation algorithm across heterogeneous imaging environments.

### 2.2. Image Acquisition and Preprocessing

LDCT examinations were performed using 64-channel or higher multidetector CT scanners, in accordance with the standardized hepatic protocol; the slice thickness ranged from 2.5 to 5 mm. Arterial-phase images were acquired approximately 30–35 s after contrast injection to capture the optimal hepatic arterial enhancement. Correspondingly, CTHA images were obtained during direct injection of the contrast media through a catheter positioned in the common or proper hepatic artery, which served as the ground truth for hypervascularity.

For data preprocessing, raw DICOM images were converted into a standardized format for deep learning integration. To ensure that the model focused exclusively on hepatic features rather than background noise, we performed liver segmentation using semi-automated software (Aview (version 1.1.40.7), Coreline Soft, Seoul, Republic of Korea). Subsequently, distinct window settings were applied to optimize the contrast visibility for each domain: a window level of 60 HU and width of 400 HU for the source domain (LDCT) and a level of 200 HU and width of 500 HU for the target domain (CTHA). Although the U-GAT-IT architecture inherently supports unpaired image-to-image translation, we implemented anatomical slice-matching for the training dataset based on the landmarks. This approach was adopted to enhance training stability and minimize geometric hallucinations during the domain adaptation process.

### 2.3. Deep Learning Architecture (U-GAT-IT)

**Figure 2:**
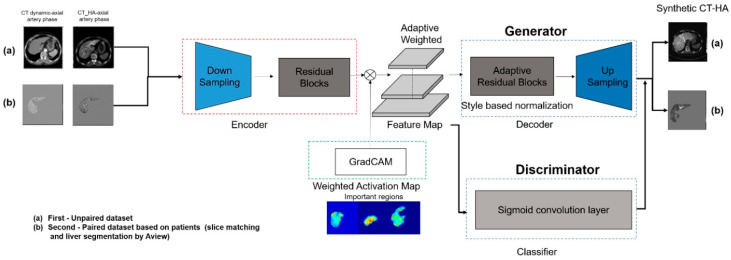
Schematic architecture of the deep learning model (U-GAT-IT). The model consists of a generator and a discriminator with an attention module. (a) The Class Activation Map (CAM) helps the model identify and focus on discriminative regions (i.e., hypervascular tumors) between the source (LDCT) and target (CTHA) domains. (b) Adaptive Layer-Instance Normalization (AdaLIN) dynamically adjusts the parameters to preserve the anatomical structure of the liver while transferring the contrast enhancement style.

We employed the U-GAT-IT architecture, which effectively mitigates the geometric distortions often observed in standard CycleGANs, while preserving the semantic content (Figure 2). The framework comprises two generators (LDCT to CTHA and vice versa) and two discriminators, incorporating two pivotal mechanisms to enhance performance.

First, the model integrates an auxiliary classifier that generates class activation maps. These maps explicitly highlight discriminative regions, specifically hypervascular HCC lesions, allowing the generator to focus on enhancing these pathological features rather than on irrelevant background textures. Second, Adaptive Layer-Instance Normalization (AdaLIN) dynamically regulates the parameters between Layer and Instance Normalization. This mechanism enables the model to flexibly control the degree of shape and texture translation, thereby preventing excessive “style transfer” that could compromise the anatomical integrity of the liver parenchyma.

Network training was governed by a composite objective function comprising four distinct losses to ensure robust image synthesis. Adversarial loss was employed to ensure that the generated images were indistinguishable from the real CTHA images, whereas cycle consistency loss enforced bidirectional mapping accuracy to preserve the original image content. Additionally, identity loss was utilized to maintain color composition, and CAM loss was implemented to guide the attention maps toward the discriminative tumor regions.

### 2.4. Image Analysis and Clinical Evaluation

#### 2.4.1. Qualitative Assessment Protocol

Image analysis was performed independently by two board-certified radiologists with 4 and 8 years’ experience in abdominal intervention and abdominal imaging, respectively. To mitigate potential bias, both readers were blinded to clinical data and the original CTHA reports throughout the evaluation process.

**Figure 3:**
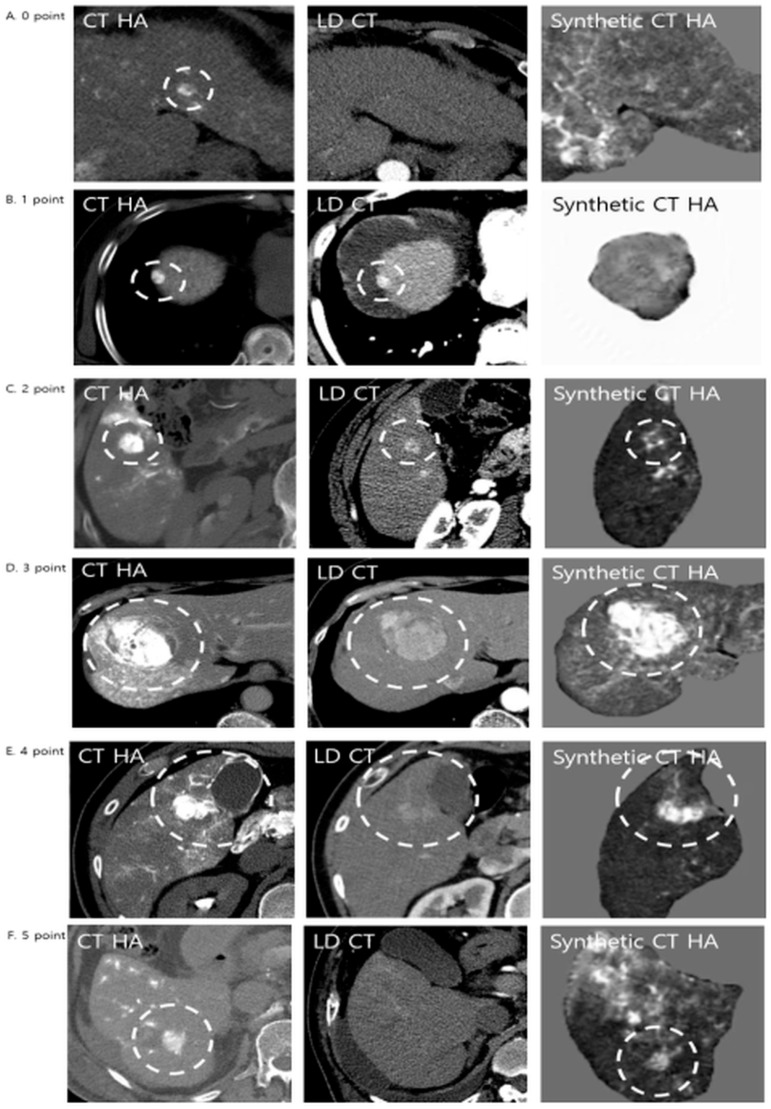
Representative examples of diagnostic performance comparing LDCT and Synthetic CTHA. Lesions were evaluated using a 5-point diagnostic value score. The white dashed circles indicate the hypervascular lesions. (**A**) Score 0: The lesion is invisible on both LDCT and Synthetic CTHA. (**B**) Score 1: The lesion is clearly visible on LDCT but lost on Synthetic CTHA (inferior). (**C**) Score 2: The lesion is visible on Synthetic CTHA but with inferior quality compared to LDCT. (**D**) Score 3: The lesion shows equivalent conspicuity on both modalities. (**E**) Score 4 (Superior): The lesion is visible on LDCT, but Synthetic CTHA shows it more clearly with higher contrast. (**F**) Score 5 (Significantly Superior): The lesion is subtle or invisible on LDCT but is clearly visualized on Synthetic CTHA, demonstrating the potential for detecting occult HCCs.

Qualitative assessment comprised three distinct components. First, lesion conspicuity was quantified using a three-point visual grading scale: grade 1 denoted lesions that were not visible; grade 2 indicated lesions visible only upon adjusting window settings; and grade 3 represented lesions clearly visible at standard settings. Second, the comparative detection value of the synthetic images relative to LDCT was evaluated using a five-point Likert scale (Figure 3). In this hierarchy, score 0 indicated that the lesion was invisible on both modalities, while scores 1 and 2 reflected instances where the synthetic image was inferior to the LDCT (lesion lost or lower quality). A score of 3 denoted an equivalent detection value. Conversely, scores 4 and 5 represented superior detection value, with score 5 specifically highlighting “occult” lesions—those invisible on LDCT but successfully visualized on synthetic CTHA. Finally, lesion detection rates were stratified by tumor diameter (<1 cm, 1–2 cm, and ≥2 cm) to assess size-dependent sensitivity.

Following the independent reviews, any inter-observer discrepancies regarding scoring or lesion detection were adjudicated through a consensus meeting, where images were jointly re-evaluated to reach a final decision.

#### 2.4.2. Quantitative Quality Assessment

Three quantitative metrics were calculated to objectively corroborate the visual findings. The contrast-to-noise ratio (CNR) was determined using the formula: (ROI_lesion − ROI_liver)/SD_liver, providing a measure of lesion distinctness. Furthermore, structural fidelity and reconstruction accuracy were evaluated using the structural similarity index measure (SSIM) and peak signal-to-noise ratio (PSNR), respectively. These metrics were subsequently analyzed across the subgroups (categorized as synthetically superior, equal, or inferior) to verify the consistency between the quantitative signal characteristics and qualitative reader preferences.

### 2.5. Statistical Analysis

Continuous variables were evaluated using non-parametric methods: the Wilcoxon signed-rank test was employed for paired comparisons, while the Kruskal–Wallis test was used for multiple group comparisons. McNemar’s test was performed to assess the differences in detection sensitivity between modalities. Furthermore, interobserver reliability for qualitative grading was examined using the McNemar–Bowker test. Statistical significance was established at a two-sided *p*-value < 0.05. All statistical computations were performed using the SPSS software (version 20.0; IBM Corp., Armonk, NY, USA).

Lesion-level analysis was primarily employed because the clinical management of HCC, including localized interventional therapies, depends on the identification of individual nodules. To ensure statistical independence, patients with diffuse HCC or uncountable multifocal lesions were excluded from the analysis.

## 3. Results

### 3.1. Diagnostic Performance

#### 3.1.1. Internal Validation Cohort

The internal validation cohort comprised 68 patients harboring a total of 139 confirmed HCC lesions (Table 2). To assess size-dependent performance, these lesions were stratified into three categories: sub-centimeter (<1 cm, *n* = 27), small (1–2 cm, *n* = 47), and medium-to-large (≥2 cm, *n* = 65).

##### Detection Rate

The application of synthetic CTHA yielded a statistically significant improvement in the detection of small hypervascular lesions. Most notably, for sub-centimeter lesions (<1 cm), the synthetic modality significantly outperformed standard LDCT, increasing the detection rate from 40.7% (11/27) to 66.7% (18/27) (*p* < 0.05). This corresponded to the successful identification of seven additional lesions that were otherwise occult on standard imaging. For intermediate-sized lesions (1–2 cm), synthetic CTHA demonstrated improved, albeit marginal, sensitivity compared with LDCT (37/47 vs. 35/47). Conversely, for lesions ≥ 2 cm, both modalities exhibited excellent sensitivity (62/65, 95.4%) with no significant performance gap, confirming that the primary clinical benefit of the model lies in detecting minute, early-stage foci.

##### Qualitative Assessment and Detection Value

Beyond the binary detection rates, visual grading analysis confirmed the superior conspicuity of the generated images. The mean visual grades for synthetic CTHA were significantly higher than those for LDCT for reviewers 1 (2.32 vs. 2.13; *p* < 0.05) and 2 (2.45 vs. 2.15; *p* < 0.05). This suggests that the generative model effectively enhances the tumor-to-background contrast, sharpening the definition even for lesions that were already visible on standard scans.

Regarding the comparative detection value, the synthetic modality provided substantial clinical benefits. Reviewers 1 and 2 assigned superior detection scores (score 4 or 5) to 37.4% (52/139) and 43.1% (60/139) of the lesions, respectively. Importantly, instances in which synthetic generation resulted in inferior image quality were infrequent. Interobserver reliability remained robust throughout the evaluation, with no statistically significant divergence in the scoring distribution (*p* > 0.05).

### 3.2. Detection Performance: External Validation

#### 3.2.1. Robustness Across Heterogeneous Environments

To verify the generalizability of the proposed model against potential domain shifts, such as variations in CT scanner manufacturers and imaging protocols, we evaluated its performance in an independent external validation cohort comprising 87 patients with 117 HCC lesions (Table 2).

##### Detection Rate

The trend toward improved sensitivity for smaller lesions, which was observed in the internal set, was consistently reproduced in the external cohort. For sub-centimeter lesions (<1 cm), synthetic CTHA demonstrated a distinct advantage, detecting 73.7% (14/19) of lesions, compared with only 57.9% (11/19) for standard LDCT. Similarly, for small lesions measuring 1–2 cm, the synthetic modality achieved a higher detection rate of 90.9% (40/44) versus 84.1% (37/44) for LDCT. Notably, for large lesions (>2 cm), synthetic CTHA achieved perfect sensitivity (54/54, 100%), whereas standard LDCT failed to identify one lesion (53/54, 98.1%). This confirms that the model not only significantly boosts the visibility of small, subtle tumors but also maintains superior reliability for overt masses over standard imaging.

##### Visual Grading and Qualitative Consistency

Visual grading analysis further corroborated the robustness of the image generation algorithm. Although the data originated from different institutions, the mean visual grades for synthetic CTHA were significantly higher than those for LDCT for both readers. Specifically, for reviewer 1, the mean grade increased from 2.47 to 2.76 (*p* < 0.05), and from 2.48 to 2.74 for reviewer 2 (*p* < 0.05). This uniform improvement underscores the model’s ability to provide sharper lesion definition and enhanced tumor-to-liver contrast, irrespective of the acquisition source.

##### Detection Value Assessment

In the comparative evaluation of clinical utility (5-point scale), the synthetic images were deemed to offer a superior detection value (score 4 or 5) in the majority of cases. Reviewers 1 and 2 assigned high scores to 59.0% (69/117) and 53.8% (63/117) of lesions, respectively. Conversely, cases in which synthetic generation resulted in inferior quality were minimal (9 and 11 cases, respectively). The inter-reviewer reliability for this external dataset was high, with no statistically significant difference in the scoring distribution (*p* > 0.05), demonstrating that the perceived clinical benefit is reproducible among different observers.

### 3.3. Quantitative Image Quality Analysis

Quantitative signal metrics provided objective corroboration of the qualitative visual assessments. In the internal validation set, the mean CNR of the lesions on synthetic CTHA was 1.85 (±1.62). Notably, subgroup analysis stratified by detection value (superior, equal, or inferior) revealed no statistically significant variation in the CNR (*p* > 0.05). This lack of variance suggests that the model generates consistent contrast enhancement across lesions, independent of baseline visibility or final detection score.

Furthermore, structural fidelity and reconstruction quality were maintained at high levels, with a mean SSIM of 0.771 (±0.063) and PSNR of 17.82 (±2.80) dB. These metrics indicate that the domain adaptation process successfully preserved the underlying anatomical details of the hepatic vessels and parenchyma, without introducing severe distortions, thereby validating the architectural robustness of the U-GAT-IT model.

### 3.4. Analysis of False Positives and Generative Artifacts

Although the synthetic modality significantly enhanced detection sensitivity, the incidence of false-positive findings necessitated rigorous scrutiny. In the internal set (68 patients), 57 false-positive nodules were identified across 42 patients (mean 1.36 per patient). Similarly, in the external set (87 patients), 39 false positives were detected across 35 patients (mean 1.11 per patient). The rate of false-positive generation did not differ significantly between the internal and external cohorts (*p* > 0.05), suggesting a stable error rate across different datasets.

#### 3.4.1. Etiology of False Positives

**Figure 4:**
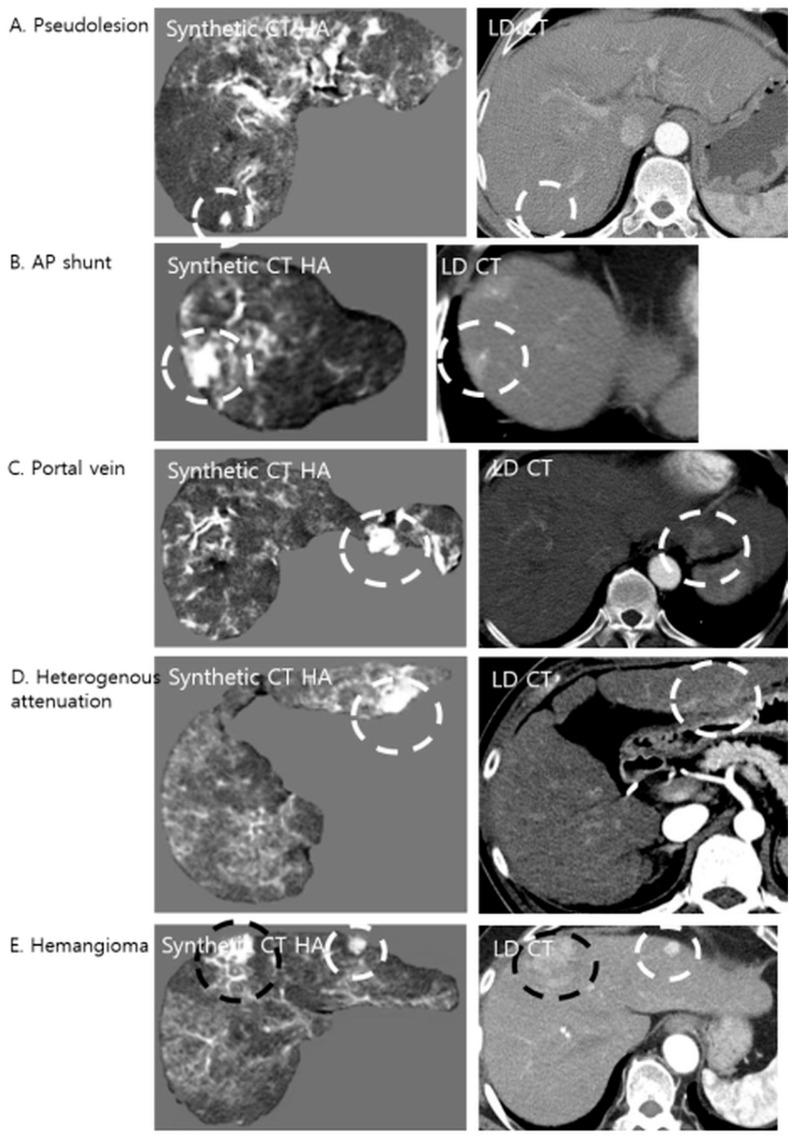
Examples of false-positive lesions on Synthetic CTHA. The algorithm may enhance non-tumorous structures, mimicking hypervascular nodules. The white dashed circles indicate pseudolesions, while the black dashed circles indicate the confirmed hypervascular hepatocellular carcinoma (HCC) lesions. (**A**) A pseudolesion generated in the absence of any enhancing structure on LDCT. (**B**) An arterioportal (AP) shunt showing early enhancement is accentuated, appearing nodular. (**C**) A cross-section of a portal vein mimicking a nodule. (**D**) Focal heterogeneous liver attenuation amplified by the model. (**E**) A flash-filling hemangioma showing enhancement patterns similar to HCC on the synthetic image.

Retrospective analysis of the corresponding LDCT images revealed the primary cause of these false positives (Figure 4). The predominant etiology was an arterioportal (AP) shunt (*n* = 47), which accounted for the majority of cases. Since AP shunts exhibit early arterial enhancement, physiologically mimicking HCC, the model, which was trained to amplify hypervascular features, accentuated these regions, rendering them nodular in appearance. Other anatomical confounders included cross-sections of the hepatic vessels (*n* = 15), focal areas of heterogeneous liver parenchyma (*n* = 14), and flash-filling hemangiomas (*n* = 2), all of which presented high attenuation values that the algorithm interpreted as potential lesions.

#### 3.4.2. Generative Artifacts (Hallucinations)

Pure generative artifacts, often termed “hallucinations,” were identified in 18 cases (10 in the internal set and 8 in the external set), where no discernible anatomical or pathological substrate existed on the original LDCT. Importantly, the incidence of these hallucinations was low and did not differ significantly between the internal (10 instances in 9 patients) and external (8 instances in 8 patients) sets (Fisher’s exact test, *p* > 0.05). Clinically, these artifacts are readily distinguishable from true HCC nodules by cross-referencing with portal venous or delayed-phase images, thereby preventing potential misdiagnosis in a practical workflow.

## 4. Discussion

### 4.1. Clinical Implications: Non-Invasive Adjunctive Tool

In this multicenter study, we developed a deep learning framework capable of generating synthetic CTHA images directly from non-invasive LDCT scans. Our findings substantiate that this synthetic approach significantly enhances the detection sensitivity for sub-centimeter hypervascular lesions (<1 cm), effectively bridging the diagnostic gap between non-invasive cross-sectional imaging and invasive gold-standard angiography.

The most pivotal clinical contribution of our study is the enhanced conspicuity of sub-centimeter lesions. Standard LDCT frequently fails to visualize these minute foci owing to subtle enhancement patterns that are obscured by the background heterogeneity of the cirrhotic liver. Our U-GAT-IT model, trained on high-contrast CTHA data, effectively learned to selectively amplify these faint arterial signals.

### 4.2. Impact on Treatment Strategies and Prognosis

The improved detection of early-stage HCCs (<1 cm) is not only a diagnostic refinement but also a critical prognostic determinant. As highlighted in recent guidelines, the therapeutic window for curative modalities—such as radiofrequency ablation or surgical resection—is strictly size-dependent [7,8,9,22]. Identifying HCCs at the sub-centimeter stage allows for curative intervention rather than palliative options such as TACE, thereby conferring a tangible survival benefit. Furthermore, in the context of interventional radiology, this synthetic tool can serve as a “virtual planning angiography.” By predicting the presence of occult lesions before the procedure, interventionalists can optimize catheter targeting and ensure complete tumor coverage, potentially reducing the rate of residual tumor recurrence.

### 4.3. Robustness and Generalizability

A distinguishing strength of this study is the rigorous external validation. Deep learning models in radiology are often plagued by “domain shift,” where an algorithm trained on data from one institution fails to generalize to another due to variations in scanner physics and acquisition protocols. However, our model demonstrated remarkable robustness in the external cohort (Guro and Ansan Hospitals), reproducing the improvements in sensitivity and reduction in false-positive rates observed in the internal set. This generalizability is likely attributable to the AdaLIN function within the U-GAT-IT architecture, which effectively harmonizes the style discrepancies between diverse datasets while meticulously preserving the semantic anatomical content.

### 4.4. Technical Consideration: GANs vs. Diffusion Models

Although recent advancements in generative AI have led to a surge in diffusion probabilistic models with high stability [23,24], GANs remain a robust and highly efficient paradigm for cross-modality medical image synthesis, particularly in resource-constrained clinical environments [25,26]. Recent studies conducted in 2024 and 2025 underscore that GAN-based frameworks, such as CycleSGAN and ICycle-GAN, continue to evolve by incorporating semantic-preserving constraints and correction networks to ensure anatomical integrity during liver image generation [27,28]. Furthermore, the computational efficiency and rapid inference capabilities of GANs provide a distinct advantage for real-time clinical deployment compared with the high iterative costs associated with diffusion models [25,29]. By leveraging an optimized GAN architecture (AdaLIN), our proposed synthetic CTHA framework achieved a balance between high-fidelity image synthesis and practical processing speed, fulfilling the rigorous requirements of pre-interventional HCC assessment.

### 4.5. Limitations and Future Directions

Our study has some limitations. First, the false-positive rate (approximately 1.1–1.3 per patient) warrants careful interpretation. This model tends to enhance any arterial hypervascularity, including benign entities such as AP shunts and hemangiomas. The theoretical frameworks proposed in 2025 suggest that such hallucinations are often intrinsic to deep neural networks used for image reconstruction, arising from the complex interplay between data discretization and network expressivity [30]. Furthermore, comparative analyses conducted in 2024 indicate that GAN architectures are specifically prone to “mode collapse” and may over-converge on dominant image features—in our case, arterial hypervascularity—thereby failing to distinguish pathological lesions from physiological mimics such as AP shunts [23]. Although diffusion models may offer improved stability with respect to these artifacts, we prioritized the computational efficiency of the GAN framework for this study [23], maintaining that these specificity tradeoffs are clinically manageable through multiphasic correlation and radiologist oversight. Moreover, in clinical practice, this specificity issue is manageable; radiologists can readily differentiate shunts from HCCs by cross-referencing portal venous or delayed-phase images, where shunts typically fade to isodensity, whereas HCCs exhibit washout. Future iterations of the model could incorporate multiphasic data inputs to autonomously reduce these false positives. Second, the retrospective design utilizing a TACE cohort implies a high pre-test probability of HCC. Validating the model in a true surveillance population with a lower disease prevalence is a necessary future step. Third, although we validated the model externally, future technical research should perform a direct head-to-head comparison with state-of-the-art diffusion models. Nevertheless, considering the current hardware constraints in many hospitals, our efficient GAN-based approach offers practical advantages for immediate clinical deployment.

## 5. Conclusions

Early detection of sub-centimeter HCC is crucial for curative treatment but remains challenging with conventional imaging. In this study, we developed and validated a deep-learning–based synthetic CTHA framework to overcome this limitation, without resorting to invasive procedures.

Our multicenter validation demonstrated that the proposed model significantly improves the detection rate of sub-centimeter HCCs compared with standard LDCT. The framework exhibited robust generalizability across different institutions, maintaining high structural fidelity and consistent contrast enhancement.

Clinically, this synthetic approach serves as a valuable “virtual planning angiography” in pre-interventional settings. Visualizing occult lesions offers an effective adjunctive tool to optimize treatment planning for high-risk patients, potentially bridging the diagnostic gap between non-invasive imaging and invasive angiography.

However, the potential for false positives, such as AP shunts, necessitates oversight by radiologists. Furthermore, it is important to note that the findings regarding the surveillance utility of synthetic CTHA are hypothesis-generating only. Future studies should focus on validating this model in surveillance cohorts and exploring advanced architectures to further refine diagnostic specificity.

## Figures and Tables

**Table 1:** Baseline Characteristics of the Study Population.

Characteristic	Internal Validation Set (*n* = 68)	External Validation Set (*n* = 87)
Age (years)	60.2 ± 10.9	61.5 ± 10.5
Sex		
Male	52 (76.5%)	63 (72.4%)
Female	16 (23.5%)	24 (27.6%)
Ascites		
Absent	54 (79.4%)	82 (94.3%)
Present	14 (20.6%)	5 (5.7%)
Tumor Burden		
Total Number of Lesions	139	117
Mean Tumor Size (cm)	3.3 ± 3.8	2.7 ± 2.7

Note: External Validation Set includes patients from Guro Hospital (*n* = 48) and Ansan Hospital (*n* = 39).

**Table 2:** Comparison of Detection Rates for Hepatocellular Carcinoma between LDCT and Synthetic CTHA in Internal and External Validation Sets.

	Internal Validation Set (*n* = 68 Patients, 139 Lesions)		External Validation Set (*n* = 87 Patients, 117 Lesions)	
Lesion Size	LDCT	Synthetic CTHA	LDCT	Synthetic CTHA
	No. (%)	No. (%)	No. (%)	No. (%)
<1 cm	22/46 (47.8%)	32/46 (69.6%) *	11/19 (57.9%)	14/19 (73.7%)
1–2 cm	76/95 (80.0%)	81/95 (85.3%)	37/44 (84.1%)	40/44 (90.9%)
≥2 cm	111/115 (96.5%)	112/115 (97.4%)	53/54 (98.1%)	54/54 (100%)
Total	209/256 (81.6%)	225/256 (87.9%)	101/117 (86.3%)	108/117 (92.3%)

Note: Data are number of detected lesions/total number of lesions (sensitivity, %). Abbreviations: LDCT, liver dynamic computed tomography; CTHA, CT during hepatic arteriography. * *p* < 0.05 compared with LDCT using McNemar’s test.

## Data Availability

The data presented in this study are available on request from the corresponding author. The data are not publicly available due to patient privacy and ethical restrictions.
